# Supplementary material for: Real-time 4D tracking of airborne virus-laden droplets and aerosols
Source: Commun Eng. 2023 Jun 19;2:41. doi: 10.1038/s44172-023-00088-x (PMC10955884; doi:10.1038/s44172-023-00088-x)
Supplement: Supplementary file 2 — Supporting Information [file 44172_2023_88_MOESM2_ESM.pdf]

Real-time 4D tracking of airborne virus-laden droplets and aerosols

Devendra Pal[1], Marc Amyot[2], Chen Liang[3], and Parisa A. Ariya[1,4*]

[1] *Department of Atmospheric and Oceanic Sciences, McGill University, 805 Sherbrooke Street West, Montreal, QC H3A 0B9, Canada.*

[2]*Department of Biological Sciences, Univerité de Montréal, Complexe des Sciences, 1375 Avenue Thérèse-Lavoie-Roux, Montréal QC, Canada H2V 0B3*

3 Department of Medicine, Division of Experimental Medicine, McGill University and Jewish General Hospital, 3755 Cote Sainte Catherine Rd., Montreal, Quebec, Canada, G3T 1 E2

[4] *Department of Chemistry, McGill University, 801 Sherbrooke Street West, Montréal, QC H3A 2K6, Canada.*

*Corresponding author: Parisa A. Ariya

Phone: (514) 398-6931 & (514) 398-3615

Fax: (514) 398-3797

E-mail: parisa.ariya@mcgill.ca

Figs. S1 to S12 ……………………………………………………………………….3-13

Tables S1 to S4………………………………………………………………………14-20

Captions for Movies S1 to S3………………………………………………………………20

Supplementary References…………………………………………………………………20

Supplementary Figures

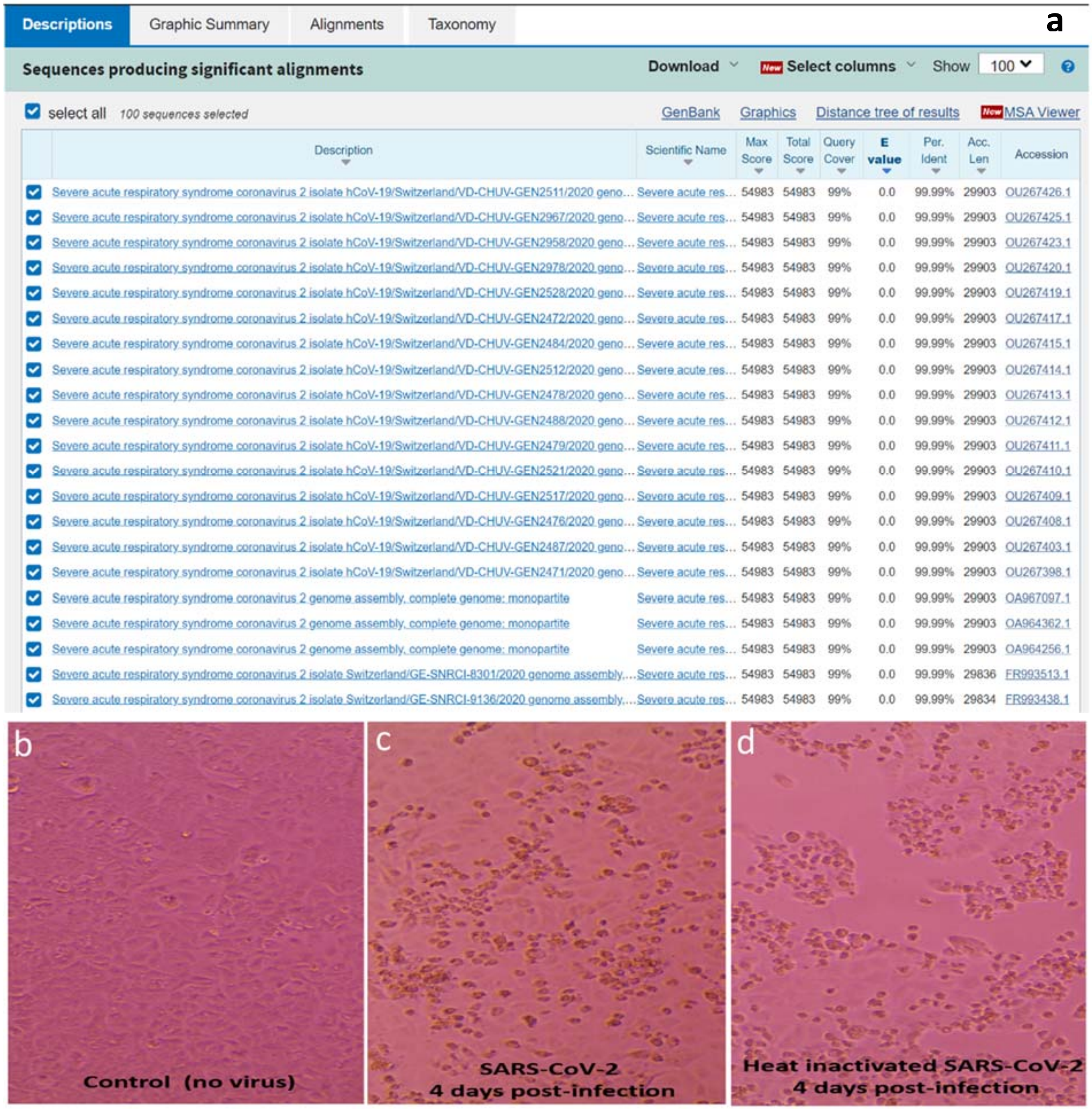

Description	Scientific Name	Max Score	Total Score	Query Cover	E value	Per. Ident	Acc. Len	Accession
Severe acute respiratory syndrome coronavirus 2 isolate hCoV-19/Switzerland/VD-CHUV-GEN2511/2020 geno...	Severe acute res...	54983	54983	99%	0.0	99.99%	29903	OU267426.1
Severe acute respiratory syndrome coronavirus 2 isolate hCoV-19/Switzerland/VD-CHUV-GEN2967/2020 geno...	Severe acute res...	54983	54983	99%	0.0	99.99%	29903	OU267425.1
Severe acute respiratory syndrome coronavirus 2 isolate hCoV-19/Switzerland/VD-CHUV-GEN2958/2020 geno...	Severe acute res...	54983	54983	99%	0.0	99.99%	29903	OU267423.1
Severe acute respiratory syndrome coronavirus 2 isolate hCoV-19/Switzerland/VD-CHUV-GEN2978/2020 geno...	Severe acute res...	54983	54983	99%	0.0	99.99%	29903	OU267420.1
Severe acute respiratory syndrome coronavirus 2 isolate hCoV-19/Switzerland/VD-CHUV-GEN2528/2020 geno...	Severe acute res...	54983	54983	99%	0.0	99.99%	29903	OU267419.1
Severe acute respiratory syndrome coronavirus 2 isolate hCoV-19/Switzerland/VD-CHUV-GEN2472/2020 geno...	Severe acute res...	54983	54983	99%	0.0	99.99%	29903	OU267417.1
Severe acute respiratory syndrome coronavirus 2 isolate hCoV-19/Switzerland/VD-CHUV-GEN2484/2020 geno...	Severe acute res...	54983	54983	99%	0.0	99.99%	29903	OU267415.1
Severe acute respiratory syndrome coronavirus 2 isolate hCoV-19/Switzerland/VD-CHUV-GEN2512/2020 geno...	Severe acute res...	54983	54983	99%	0.0	99.99%	29903	OU267414.1
Severe acute respiratory syndrome coronavirus 2 isolate hCoV-19/Switzerland/VD-CHUV-GEN2478/2020 geno...	Severe acute res...	54983	54983	99%	0.0	99.99%	29903	OU267413.1
Severe acute respiratory syndrome coronavirus 2 isolate hCoV-19/Switzerland/VD-CHUV-GEN2488/2020 geno...	Severe acute res...	54983	54983	99%	0.0	99.99%	29903	OU267412.1
Severe acute respiratory syndrome coronavirus 2 isolate hCoV-19/Switzerland/VD-CHUV-GEN2479/2020 geno...	Severe acute res...	54983	54983	99%	0.0	99.99%	29903	OU267411.1
Severe acute respiratory syndrome coronavirus 2 isolate hCoV-19/Switzerland/VD-CHUV-GEN2521/2020 geno...	Severe acute res...	54983	54983	99%	0.0	99.99%	29903	OU267410.1
Severe acute respiratory syndrome coronavirus 2 isolate hCoV-19/Switzerland/VD-CHUV-GEN2517/2020 geno...	Severe acute res...	54983	54983	99%	0.0	99.99%	29903	OU267409.1
Severe acute respiratory syndrome coronavirus 2 isolate hCoV-19/Switzerland/VD-CHUV-GEN2476/2020 geno...	Severe acute res...	54983	54983	99%	0.0	99.99%	29903	OU267408.1
Severe acute respiratory syndrome coronavirus 2 isolate hCoV-19/Switzerland/VD-CHUV-GEN2487/2020 geno...	Severe acute res...	54983	54983	99%	0.0	99.99%	29903	OU267403.1
Severe acute respiratory syndrome coronavirus 2 isolate hCoV-19/Switzerland/VD-CHUV-GEN2471/2020 geno...	Severe acute res...	54983	54983	99%	0.0	99.99%	29903	OU267398.1
Severe acute respiratory syndrome coronavirus 2 genome assembly, complete genome: monopartite	Severe acute res...	54983	54983	99%	0.0	99.99%	29903	OA967097.1
Severe acute respiratory syndrome coronavirus 2 genome assembly, complete genome: monopartite	Severe acute res...	54983	54983	99%	0.0	99.99%	29903	OA964362.1
Severe acute respiratory syndrome coronavirus 2 genome assembly, complete genome: monopartite	Severe acute res...	54983	54983	99%	0.0	99.99%	29903	OA964256.1
Severe acute respiratory syndrome coronavirus 2 isolate Switzerland/GE-SNRCI-8301/2020 genome assembly,...	Severe acute res...	54983	54983	99%	0.0	99.99%	29836	FR993513.1
Severe acute respiratory syndrome coronavirus 2 isolate Switzerland/GE-SNRCI-9136/2020 genome assembly,...	Severe acute res...	54983	54983	99%	0.0	99.99%	29834	FR993438.1

Figure S1. SARS-CoV-2 samples were obtained from the Medicine Department at McGill University. (a) BLASTN, using the betacoronavirus genomic database, is the result of the sequenced genome of the SARS-CoV-2 sample. The GenBank ID for the sequence is MN908947.3. (c-d) SARS-CoV-2 sample images were taken with a 10X magnification AMG Evos XL core microscope at the Medicine Department, McGill University. The scale/colour bar was unavailable because the technician did not operate the instrument with the scale/colour bar enable conditions.

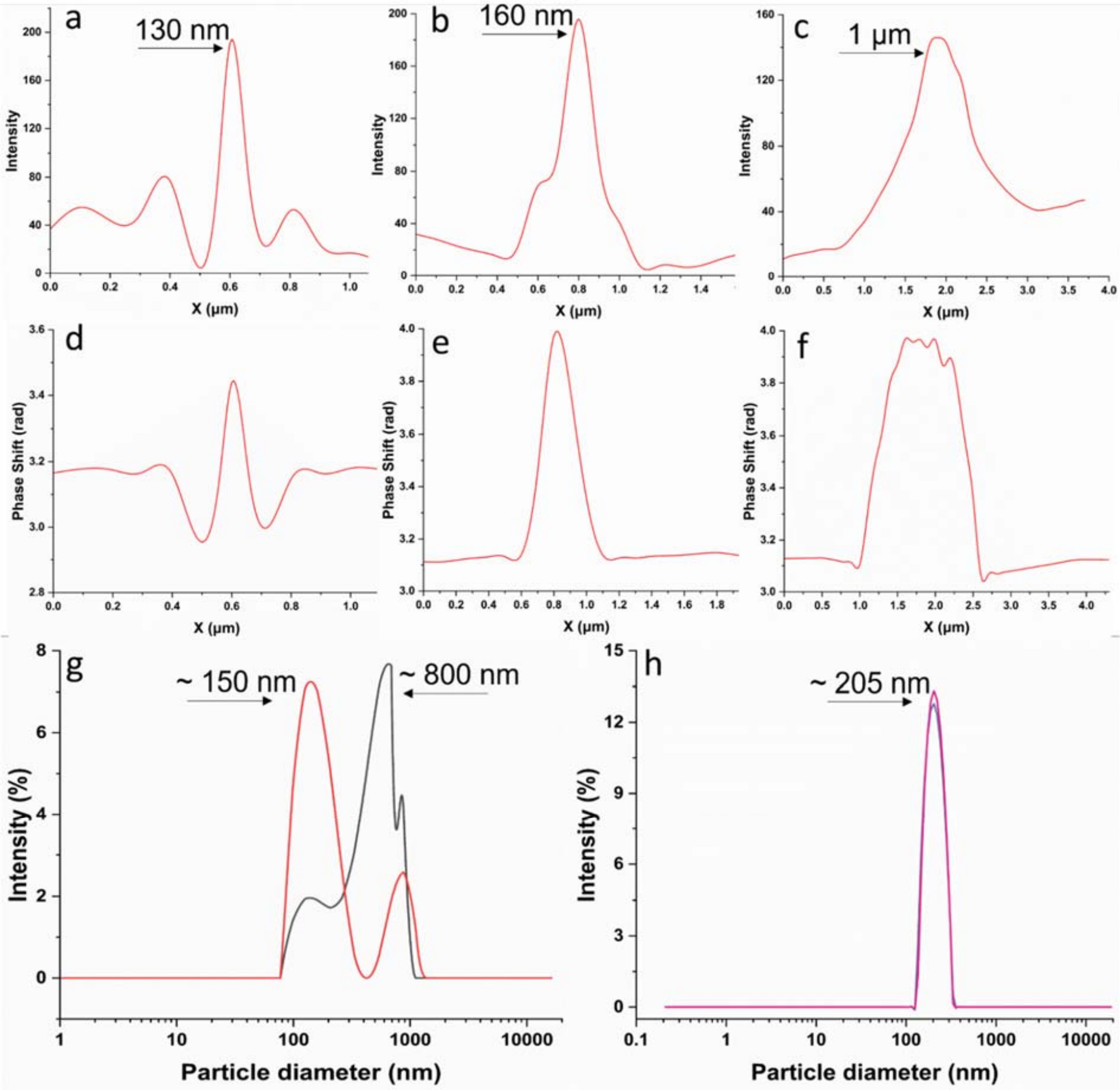

Figure S2 Intensity and phase profiles of MS2 in aqueous mode. (a-f) Intensity and phase profiles across the particle crosscuts (Fig 1c), respectively. The size of the particles is represented in terms of the full width half maximum (FWHM). (g) MS2 particle size distribution obtained by a particle sizer analyzer (PSA, Anton Paar) matched with the size obtained by nano-DIHM. (h) Calibration curve of PSA with 200 nm PSL spheres.

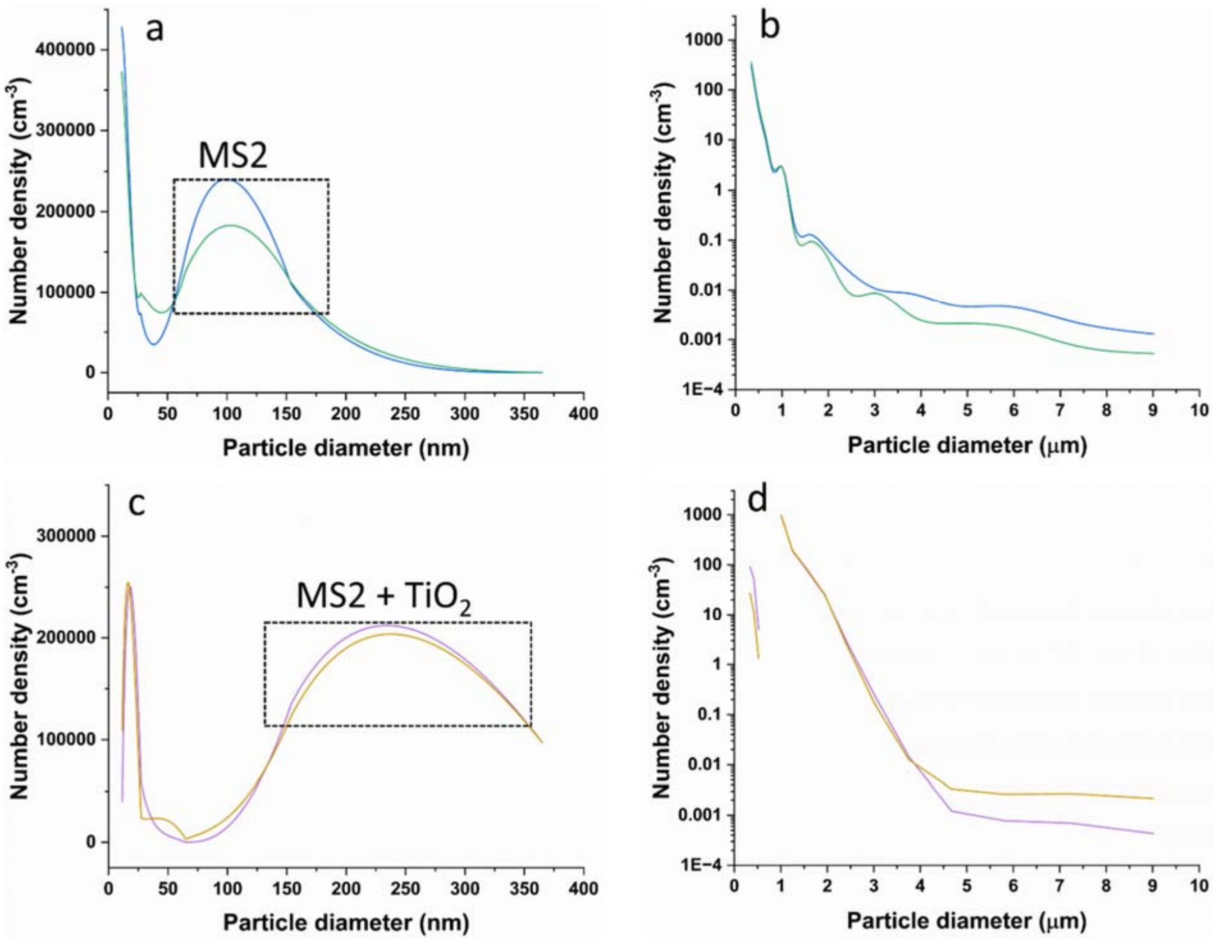

Figure S3 Size distribution of measurement of airborne MS2 bacteriophages. obtained by (a-b) Airborne MS2 particle size distribution obtained by the Scanning Mobility Particle Sizer (SMPS) and Optical Particle Sizer (OPS), respectively. (c-d) size distribution of mixed samples of MS2 and TiO_2. The two-colored line corresponds to two repetitions of an experiment.

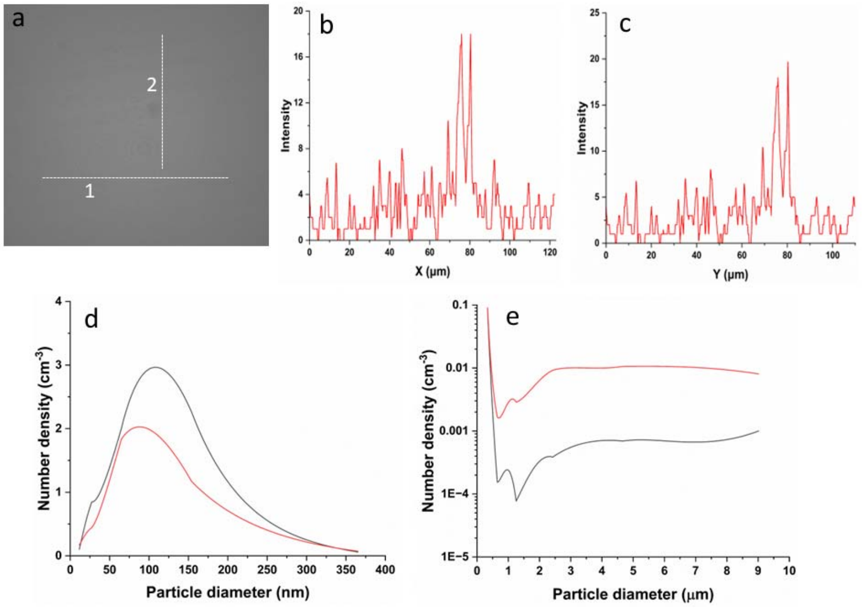

Figure S4 Nano-DIHM control experiment. (a) Background intensity hologram recorded for zero air. (b) Intensity profile across the crosscut of horizontal line 1. (c) Intensity profile along vertical line 2. (d, e) Particle size distribution of zero air and the air with a HEPA filter. The total particle count was < 3 $\#.cm^{-3}$.

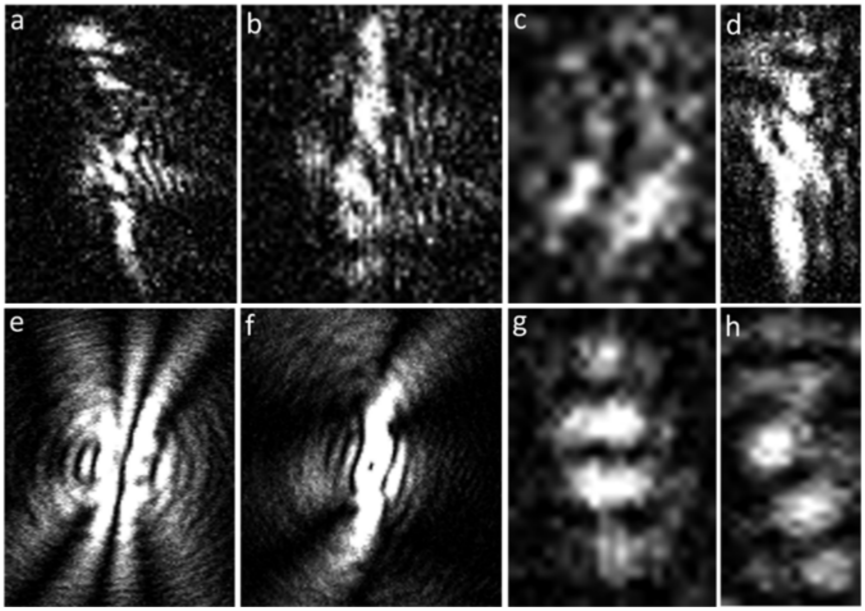

Figure S5 Automated detection of MS2- and UVB-exposed MS2 particles using Stingray software. (a-d) Shape and morphology of MS2 particles and (e-h) shape and morphology of UVB-exposed MS2 particles. Nano-DIHM clearly shows the change in the structure of MS2 and successfully determines the UV impact.

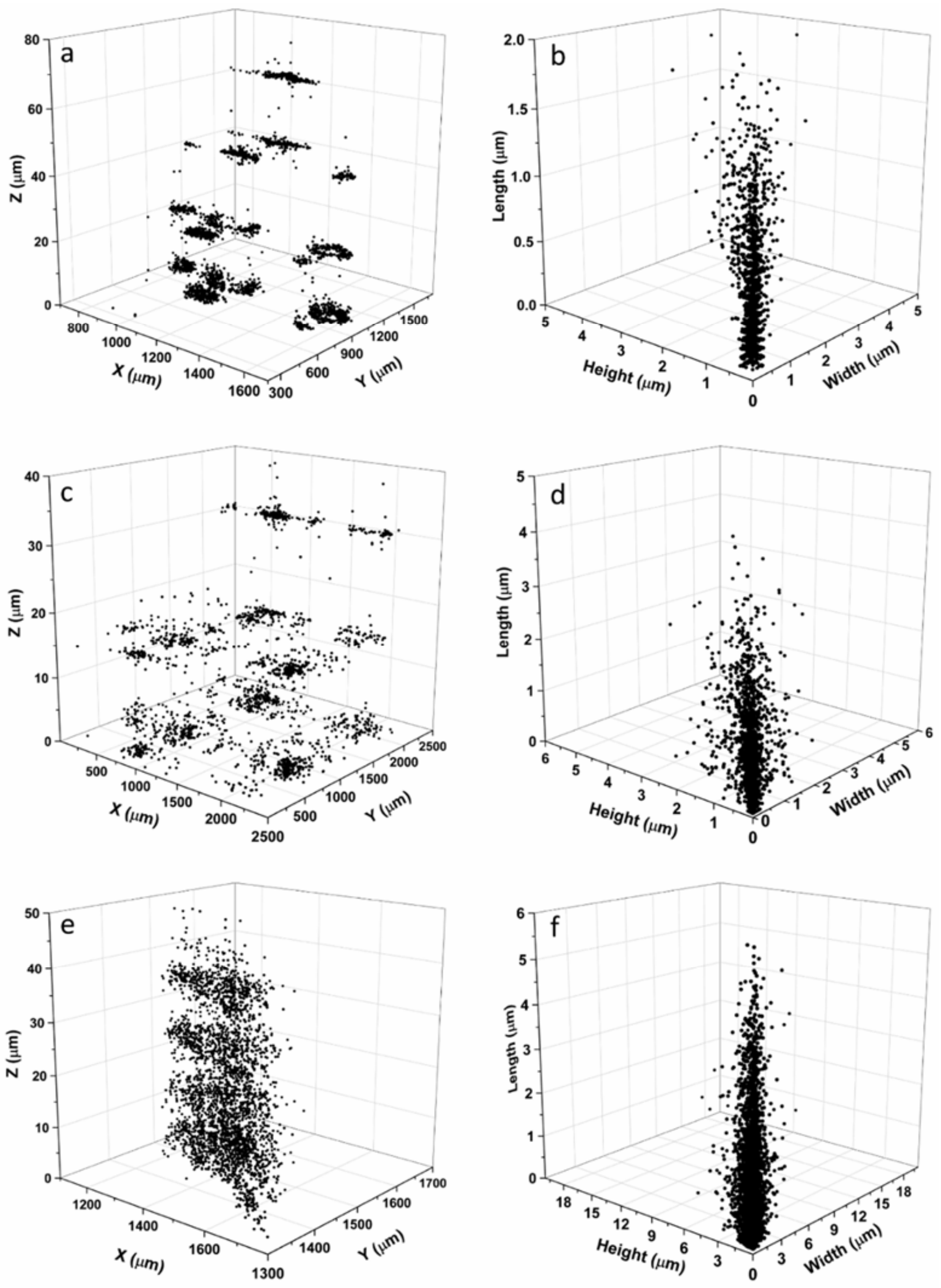

Figure S6 3D orientation and size distribution of bacteriophage MS2 viruses. (a-b) Orientation and size distribution of airborne MS2 particles (dry aerosols). (c-d) Orientation and size distribution of a mixture of MS2 and TiO2 particles. (e-f) Orientation and size distribution of viral MS2 varus-laden droplets.

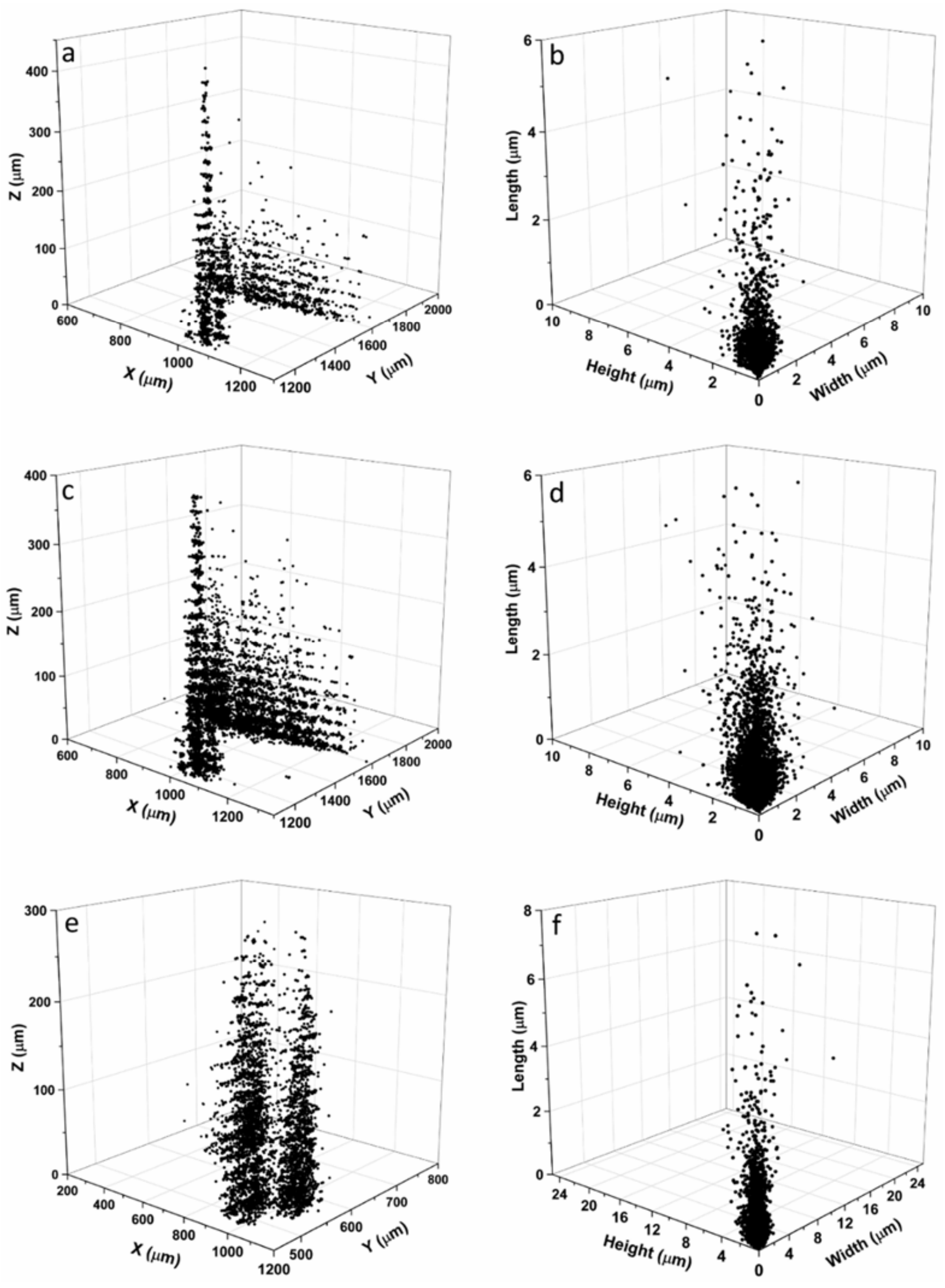

Figure S7 3D orientation and size distribution of SARS-CoV-2 viral droplets at different velocities. (a-b) Orientation and size distribution of SARS-CoV-2 viral droplets at 10 m/second, (c-d) 20 m/second and (e-f) 30 m/second.

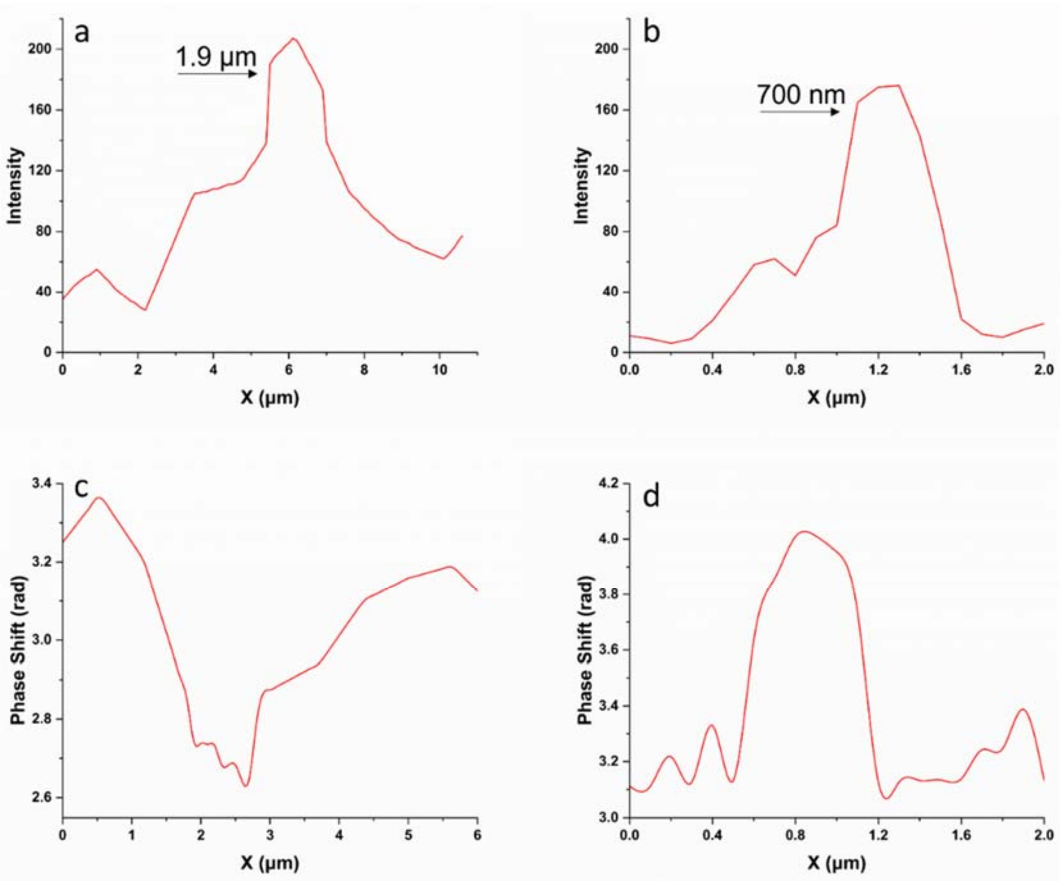

Figure S8 Intensity and phase cross-section of SARS-CoV-2 viruses in aqueous mode. (a-b) Intensity profiles across the particle crosscuts. (c-d) Phase profiles across the particle crosscuts. The intensity and phase profiles indicate the SARS-CoV-2 viral particle size from nano- to microsized.

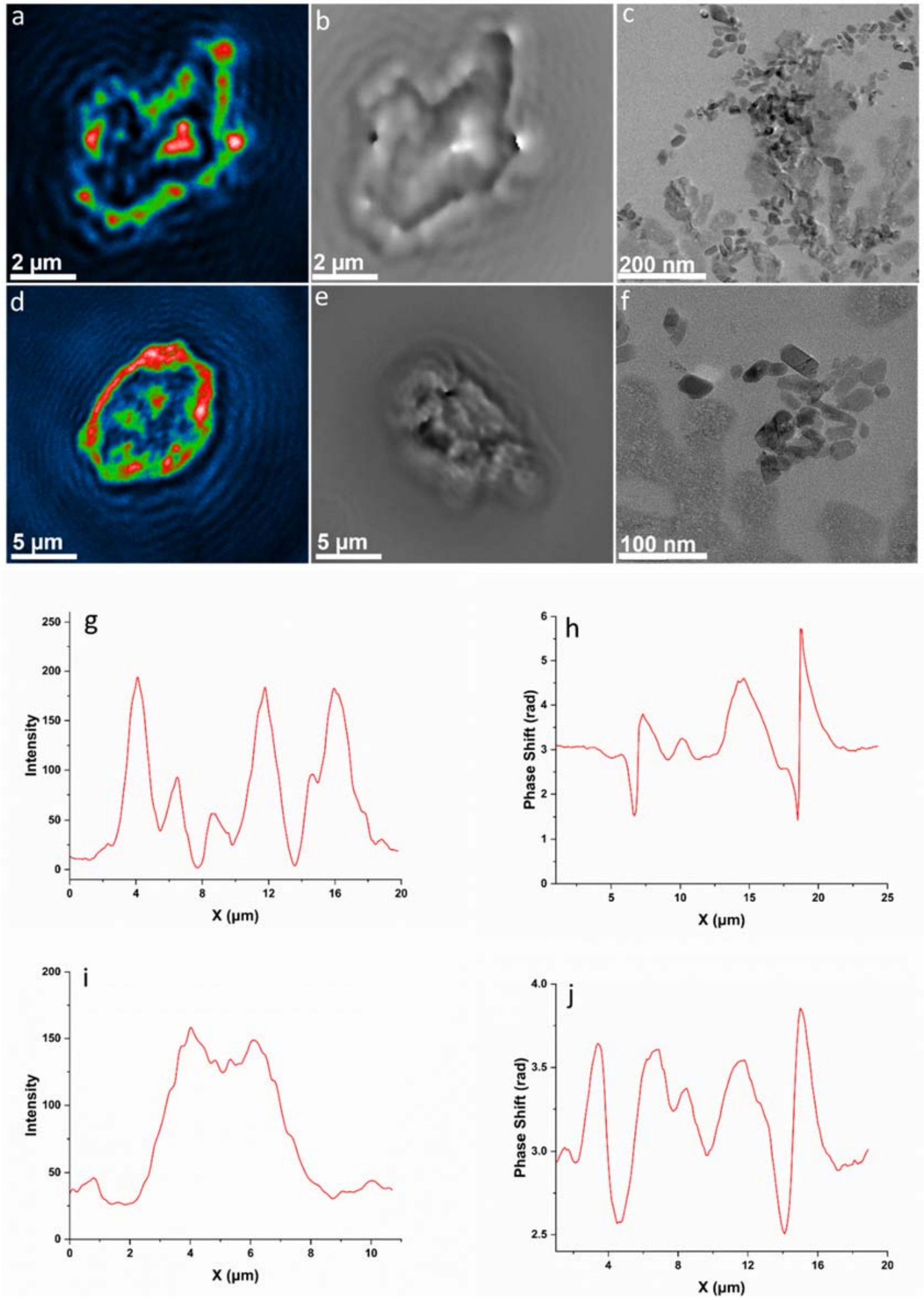

Figure S9 TiO_2 coating impact on MS2. (a-b) Intensity reconstruction of TiO2-coated MS2 viruses and (d, e) phase reconstruction of identical particles. (c, f) High-resolution

electron microscopy images, (g, i) intensity response, and (h, j) particle phase response.

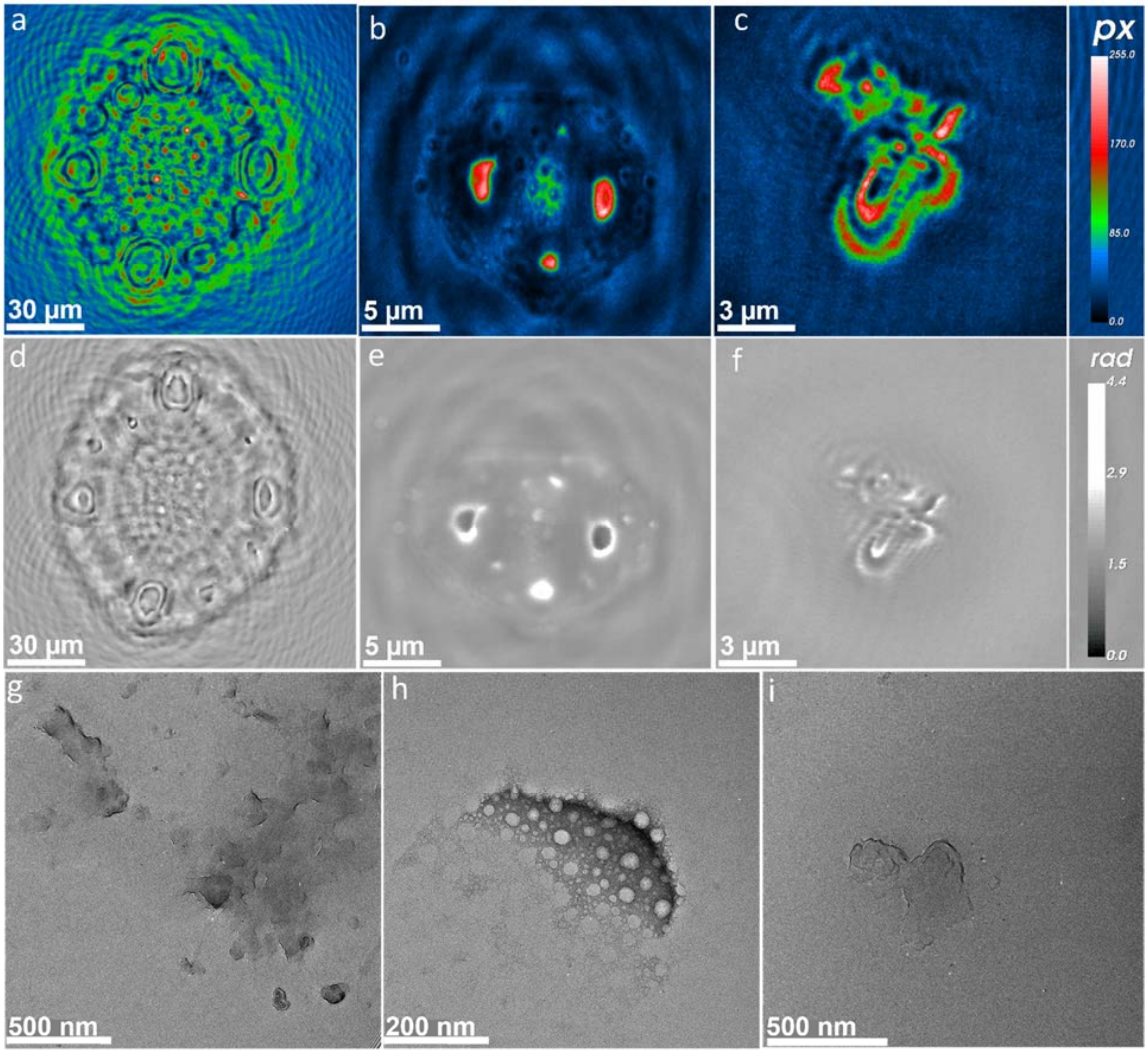

Figure S10 Olive oil coating impact on MS2. (a-c) Intensity reconstruction of olive oil-coated MS2 viruses and (d- e) phase reconstruction of identical particles. (g-i) High-resolution electron microscopy images. The shape of the oil-coated particles obtained by nano-DIHM is identical to that obtained by S/TEM.

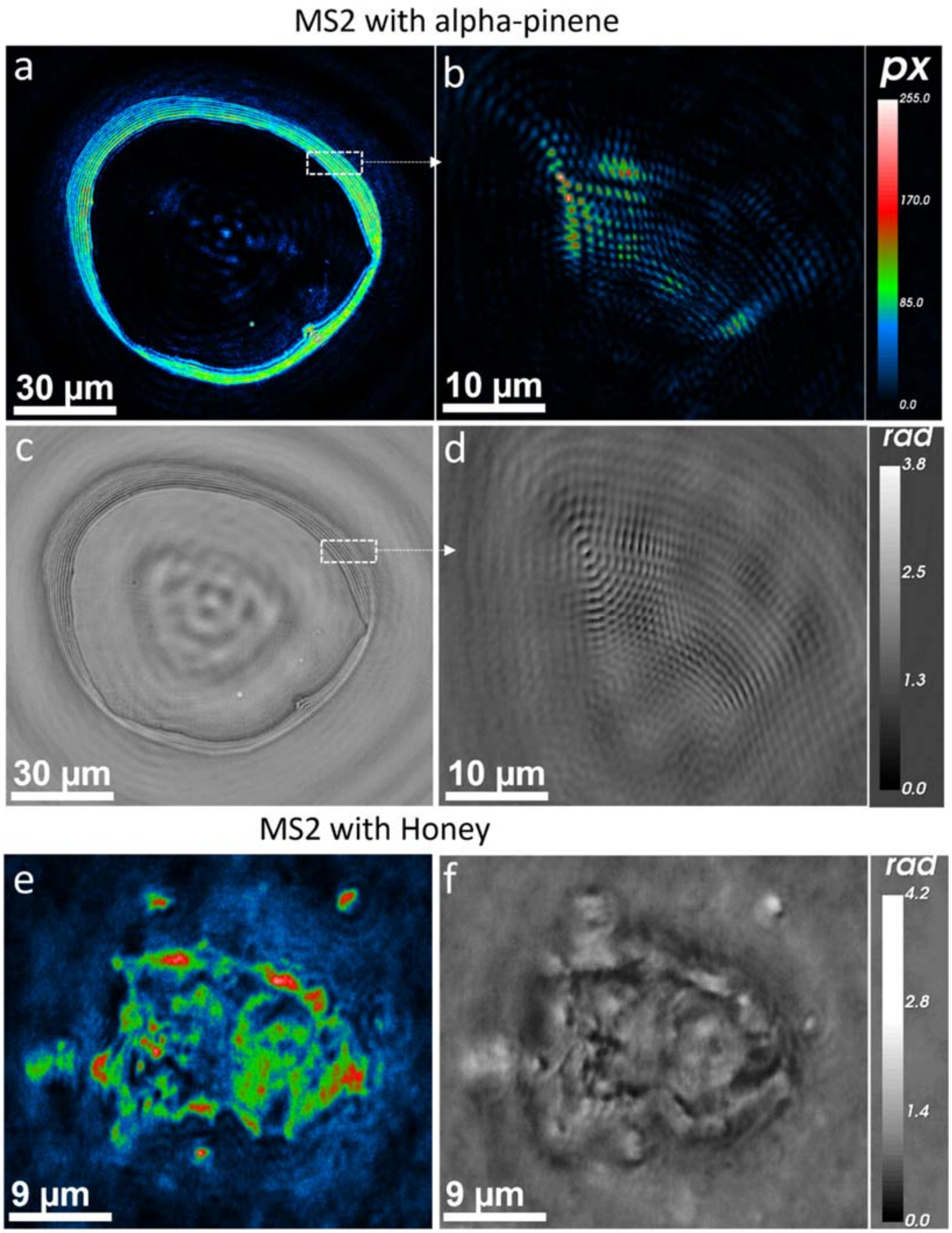

Figure S11 Natural organic coating material coating impact on MS2. (a-b) Intensity reconstruction of alpha-pinene-coated MS2 viruses and (c-d) phase reconstruction of identical particles. (e-f) Intensity and phase responses of natural honey-coated MS2 particles.

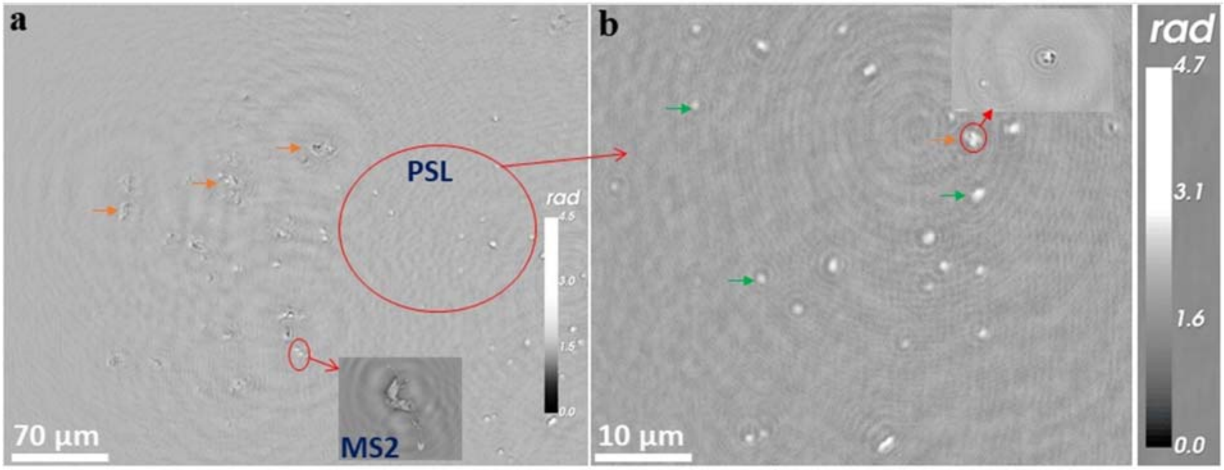

Figure S12 Deciphering MS2 from a mixed sample of MS2 and PSL. (a-b) Phase images of MS2 mixed particles with PSL. The red arrow in panel (a) indicates MS2, and the green arrow in panel (b) indicates PSL.

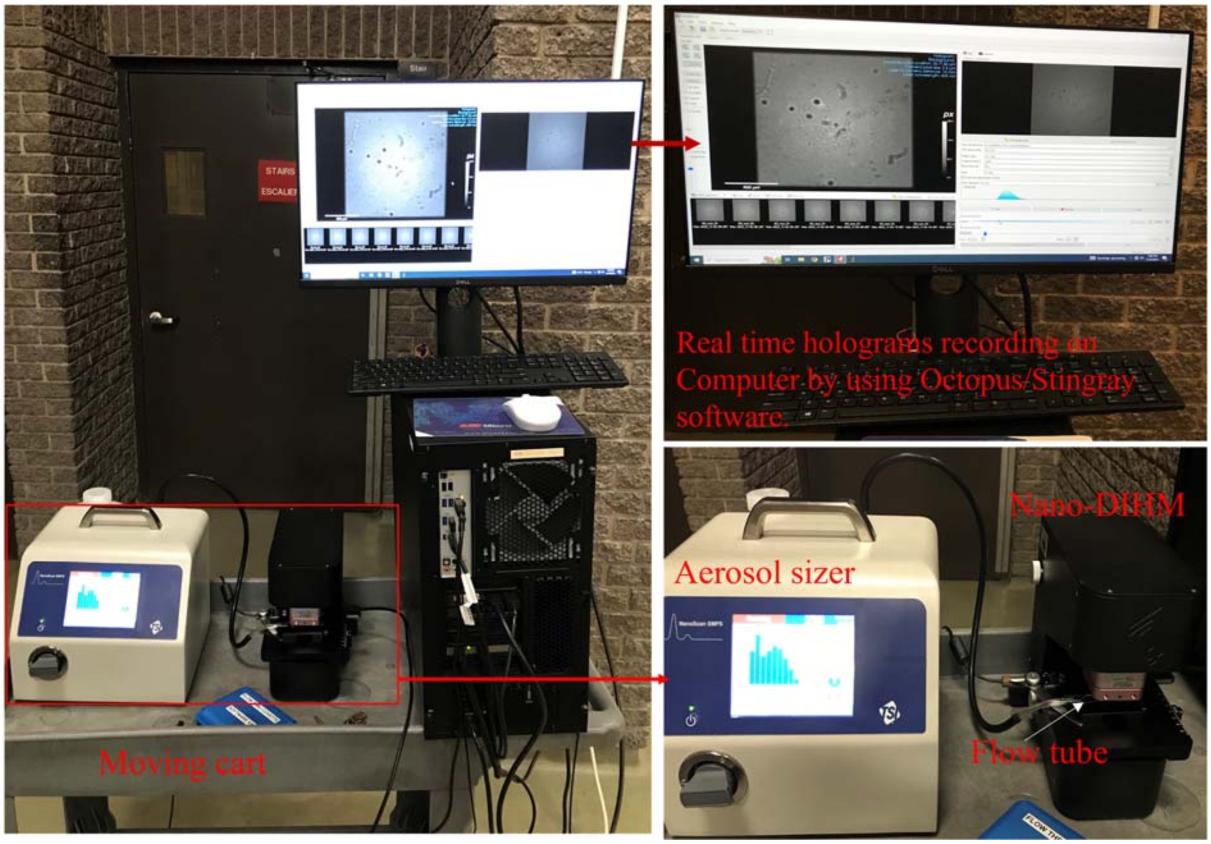

Figure S13 Portable Nano-DIHM setup at moving cart. The experimental demonstration at Burnside Hall, McGill University. Photo credit: Devendra Pal, iPhone7.

Supplementary Tables

Table S1 SARS-CoV-2 virus detection techniques based on polymer chain reaction, optical-based and industrialized techniques

**Polymer chain reaction-based techniques to detect SARS-CoV-2**		
**Sample type, collection method**	**Detection technique**	**Reference**
Solid/particulate, impactor, filters	ddPCR	(*1*)
Solid/particulate, NIOSH sampler	qPCR, RNA	(*2*)
Solid/particulate, NIOSH sampler	qRT–PCR	(*3*)
Solid/particulate, NIOSH sampler with a gelatin filter (<1 μm)	Mean qRT–PCR	(*4*)
Solid/particulate, gelatin filter, PC filter, SASS 3100	qRT–PCR	(*5*)
	Cryo-EM	(*6*)
	Cryo-EM	(*7*)
RNA transcripts	RT–PCR	(*8*)
Blood	ELISA	(*9*, *10*)
Throat swabs	RT-LAMP	(*11*)
Synthetic RNA	RPA/SHERLOCK assay	(*12*)
Nasopharyngeal, oropharyngeal swabs	RT-LAMP/Cas12 DETECTR assay	(*13*)
Nasopharyngeal swabs	FET	(*14*)
Membrane-engineered Vero cells	Cell-based potentiometric biosensor	(*15*)
Synthetic RNA	Plasmonic photothermal (PPT) enhanced LSPR	(*16*)
**Optical methods for SARS-CoV-2 detection and investigation**		
**Optical approach**	**Virus**	**References**
SERS	SARS-CoV-2	(*17*)
SM-SERS	SARS-CoV-2	

SERS	Spike protein of SARS-CoV-2	(*18*)
Fluorescence	Coronavirus diagnosis	(*19*)
SPR	SARS-CoV-2	(*20*)
SPR	SARS-CoV-2	(*21*)
LSPR	SARS-CoV-2	(*16*)
SPR	SARS-CoV-2	(*22*)
SPR	SARS-CoV-2	(*23*)
SERS, SPR	(COVID-19) (SARS-CoV-2)	(*24*)
Colorimetric assay	SARS-CoV-2	(*25*)
Holography Microscopy	COVID-19 from red blood cell	(*26*)
Holography Microscopy	SARS-CoV-2 antibody	(*27*)
Holography Microscopy	SARS-CoV-2	(*28*)
Holography Microscopy	SARS-CoV-2	(*29*)
SERS	SARS-CoV-2	(*30*)
Holography Microscopy	SARS-CoV-2	(*31*)
**Industrialized techniques based on optical methods for SARS-CoV-2 detection**		
**Technique**	**Industry**	**Reference**
SERS	Samsung Strategy and Innovation Center	(*32*)
Raman	Botanisol Analytics	(*33*)
Raman	BioMark Diagnostics Inc.	(*34*)
Raman	Advanced Nano Technologies	(*35*)
LSPR	LambdaGen corporation	(*36*)
SPR	Creative Biostructure Coronavirus	(*37*)
FTIR	Todos Medical Ltd	(*38*)
Planar Waveguide	LightDeck Diagnostics	(*39*)
Holography	Virolens	(*40*)

Table S2 Detailed experimental parameters for the matrices used. The hologram size was 2048 * 2048 pixels, and the camera pixel size was 5.5 µm during each experiment.

Sample matrices	Sample mode	Flow rate (L/min)
**Dynamic**		
**SARS-CoV-2**	Sneezing (aerosol, droplet)[1]	2.5, 1.5, 0.5 ml/min
**MS2**	Moist droplet[2]	0.5, 0.25 ml/min
**MS2**	Airborne (dry aerosols)[3]	1.7, 0.7
**MS2+TiO_2**	Airborne (dry aerosols)	1.7, 0.7
**TiO_2**	Airborne (dry aerosols)	1.7,0.7
**100 nm PSL**	Airborne (dry aerosols) *	1.7,0.7
**200 nm PSL**	Airborne (dry aerosols) *	1.7,0.7
**Stationary**		
**SARS-CoV-2**	Deposition[4]	Water
**SARS-CoV-2 RNA**	Deposition	Water
**MS2**	Deposition	Water
**MS2**	Deposition	Water
**SARS-CoV-2 + SARS-CoV-2 RNA + TiO_2**	Deposition	Water
**SARS-CoV-2 + Iron Oxide**	Deposition	Water
**MS2+TiO_2**	Deposition	Water
**MS2+PSL**	Deposition	Water
**Olive oil**	Deposition	Water
**Honey**	Deposition	Water
**Alpha-pinene**	Deposition	Water
**Olive oil + MS2**	Deposition	Water
**Honey + MS2**	Deposition	Water
**Alpha-pinene + MS2**	Deposition	Water

1. MS2 Bacteriophage samples aerosolized with C-flow atomizer and aerosol stream passed through two diffusion dryers before passing to nano-DIHM sample volume (flow tube cuvette) and SMPS/OPS. The humidity was less than 4%.
2. A bubbler was used to generate moist droplets of MS2 bacteriophage. The moist droplets directly passed through a flow tube cuvette during nano-DIHM. No dryer was used.
3. A mixed solution of MS2 with TIO2 was aerosolized, and aerosol steam was passed through a diffusion dryer.
4. SARS-CoV-2 virus droplets were generated by using a c-flow atomizer. The droplets directly passed through a flow tube cuvette into the nano-DIHM sample volume. This experiment was modeled with sneezing/coughing velocities.

Table S3 Physical characteristics of SARS-CoV-2, MS2, and other materials calculated from DIHM holograms using Stingray software. Automated physical characteristics of SARS-CoV-2, MS2, and several natural and synthetic materials in dynamic and stationary modes over 31.25 ms. The detailed automation processes of the virus and particle detection and classification procedures are discussed in the Methods section. These images are reconstructed images of objects/particles that show the shape/morphology of particles. * shows clusters.

Image	Size (µm)	Edge gradient	Roughness	Surface area (µm^2)
**SARS-CoV-2**				
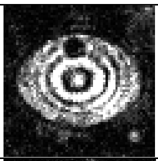	1.35	90.90	1.12	31.80
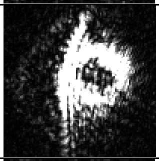	12.74*	91.03	1.55	48.66
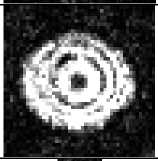	1.24	90.64	1.06	28.41
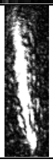	0.14	101.63	1.22	8.55
**MS2 bacteriophage**				
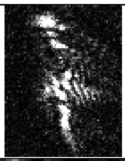	4.33	108.62	1.67	11.15
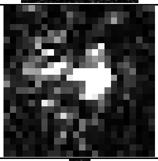	0.92	109.23	1.34	6.27
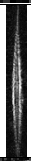	0.12	107.38	1.61	19.24
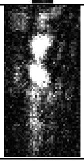	1.72	103.98	1.43	11.54
**MS2 bacteriophage (UVB)**				

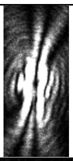	2.37	56.44	2.12	18.06
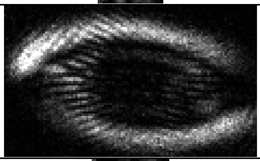	0.58	64.23	1.99	13.74
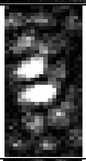	1.77	76.08	1.33	8.85
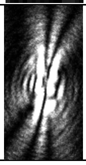	2.25	64.53	2.34	20.59
**PSL**				
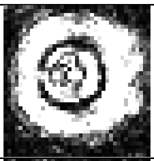	0.36	104.97	1.91	14.80
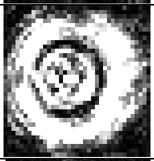	0.37	105.02	1.81	15.23
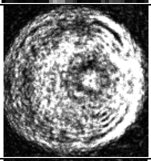	0.94	108.37	1.90	57.99
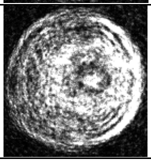	0.96	97.76	2.01	58.86
**Iron oxides (Fe_2O_3)**				
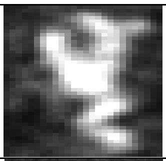	2.11	26.66	1.21	7.08
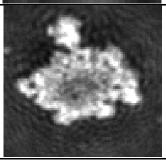	5.76	27.46	1.31	21.18

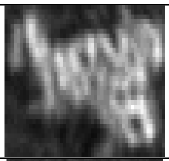	10.61*	27.51	1.27	12.05
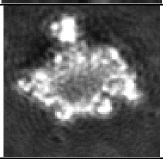	5.43	28.32	1.25	20.99
**Olive oil ($C_{88}H_{164}O_{10}$)**				
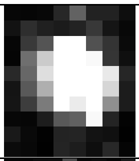	0.87	106.19	1.01	6.93
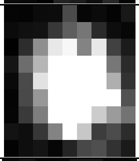	1.0	108.43	1.01	5.26
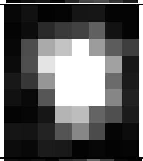	0.75	97.40	1.01	5.73
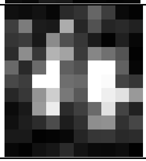	0.79	126.27	1.06	7.46

Table S4 3D size distribution of SARS-CoV-2 particles in the droplet phase with different velocities. Previous studies for droplet sizes based on a transmission mode.

**Droplet size (µm)**	**Droplet generation**	**Measurement techniques**	**References**
0.8-5.5	Droplet generation through speaking to whispered counting	Aerosol particle sizer and scanning mobility particle sizer	(*41*)
9.8-14	Cough data	Liquid impaction	(*42*)
8.1	Sneeze data	Liquid impaction	(*42*)
0.63-2.4	Cough/influenza patient	Laser aerosol particle spectrometer	(*43*)
1.6, 1.7 and 123	Aerosols disease (Bronchiolar/Laryngeal/Oral)	Aerodynamic particle sizer and droplet deposition analysis	(*44*)
7-123	Cough and sneeze	High-speed photographic technique	(*45*)

1-100	Cough and sneeze	Sample collection by using impactor	
< 1	Cough and sneeze via coxsackievirus A	Solid impaction	(*46*)
85% drops less than < 1	Cough	Solid impaction/Optical particle counter	(*47*)
Majority of respirable size	Cough	Aerosol sampling system and solid impaction	(*48*)
0.15-0.19	Breathing	Optical particle counter	(*49*)
0.62-8.35	Cough	Aerodynamic particle sizer scanning mobility particle sizer	(*50*)
< 1 (87%)	Influenza cough	Optical particle counter	(*51*)
13.5	Cough and speech	Interferometric Mie imaging technique	(*52*)

Supplementary Movies

Movie S1

Movie 1 presents the dynamic trajectories of MS2 viral droplets (sneezing modeled) in moving air.

Movie S2

Movie 2 displays the trajectories of dry MS2 aerosols in moving air.

Movie S3

Movie 3 presents the dynamic trajectories of SARS-CoV-2 viral droplets (sneezing model) in moving air.

Supplementary References

1. Y. Liu *et al.*, Aerodynamic analysis of SARS-CoV-2 in two Wuhan hospitals. *Nature* **582**, 557-560 (2020).
2. P. Y. Chia *et al.*, Detection of air and surface contamination by SARS-CoV-2 in hospital rooms of infected patients. *Nature communications* **11**, 1-7 (2020).
3. R. A. Binder *et al.*, Environmental and aerosolized severe acute respiratory syndrome coronavirus 2 among hospitalized coronavirus disease 2019 patients. *The Journal of infectious diseases* **222**, 1798-1806 (2020).
4. J. L. Santarpia *et al.*, The Infectious Nature of Patient-Generated SARS-CoV-2 Aerosol. medRxiv 2020.07. 13.20041632. *Retrieved from doi: https://doi. org/10.1101/2020.07* **13**, (2020).
5. N. Dumont-Leblond *et al.*, Low incidence of airborne SARS-CoV-2 in acute care hospital rooms with optimized ventilation. *Emerging microbes & infections* **9**, 2597-2605 (2020).

6. A. M. Ismail, A. A. Elfiky, SARS-CoV-2 spike behavior in situ: a Cryo-EM images for a better understanding of the COVID-19 pandemic. *Signal Transduction and Targeted Therapy* **5**, 252 (2020).
7. D. Wrapp *et al.*, Cryo-EM structure of the 2019-nCoV spike in the prefusion conformation. *Science* **367**, 1260-1263 (2020).
8. V. M. Corman *et al.*, Detection of 2019 novel coronavirus (2019-nCoV) by real-time RT-PCR. *Eurosurveillance* **25**, 2000045 (2020).
9. A. Krüttgen *et al.*, Comparison of the SARS-CoV-2 Rapid antigen test to the real star Sars-CoV-2 RT PCR kit. *Journal of virological methods* **288**, 114024 (2021).
10. R. Lassaunière *et al.*, Evaluation of nine commercial SARS-CoV-2 immunoassays. *MedRxiv*, (2020).
11. Y. Huang *et al.*, SARS-CoV-2 viral load in clinical samples from critically ill patients. *American journal of respiratory and critical care medicine* **201**, 1435-1438 (2020).
12. F. Zhang, O. O. Abudayyeh, J. S. Gootenberg, A protocol for detection of COVID-19 using CRISPR diagnostics. *A protocol for detection of COVID-19 using CRISPR diagnostics* **8**, (2020).
13. J. P. Broughton *et al.*, CRISPR–Cas12-based detection of SARS-CoV-2. *Nature biotechnology* **38**, 870-874 (2020).
14. G. Seo *et al.*, Rapid detection of COVID-19 causative virus (SARS-CoV-2) in human nasopharyngeal swab specimens using field-effect transistor-based biosensor. *ACS nano* **14**, 5135-5142 (2020).
15. S. Mavrikou, G. Moschopoulou, V. Tsekouras, S. Kintzios, Development of a portable, ultra-rapid and ultra-sensitive cell-based biosensor for the direct detection of the SARS-CoV-2 S1 spike protein antigen. *Sensors* **20**, 3121 (2020).
16. G. Qiu *et al.*, Dual-functional plasmonic photothermal biosensors for highly accurate severe acute respiratory syndrome coronavirus 2 detection. *ACS nano* **14**, 5268-5277 (2020).
17. R. Yan *et al.*, Structural basis for the recognition of SARS-CoV-2 by full-length human ACE2. *Science* **367**, 1444-1448 (2020).
18. H. Jinglin *et al.*, Detection of spike protein of SARS-CoV-2 by surface enhanced Raman spectroscopy. *强激光与粒子束* **32**, 069001-069001-069001-069002 (2020).
19. B. W. Editors, Optical method could cut Coronavirus diagnosis time to 15 minutes. (2020).
20. Q. Wang *et al.*, Structural and functional basis of SARS-CoV-2 entry by using human ACE2. *Cell* **181**, 894-904. e899 (2020).
21. J. Shang *et al.*, Structural basis of receptor recognition by SARS-CoV-2. *Nature* **581**, 221-224 (2020).
22. A. Djaileb *et al.*, A rapid and quantitative serum test for SARS-CoV-2 antibodies with portable surface plasmon resonance sensing. (2020).
23. J. E. Sanchez *et al.*, Detection of SARS-CoV-2 and its S and N proteins using surface enhanced Raman spectroscopy. *Rsc Advances* **11**, 25788-25794 (2021).
24. F. Cui, H. S. Zhou, Diagnostic methods and potential portable biosensors for coronavirus disease 2019. *Biosensors and bioelectronics* **165**, 112349 (2020).
25. P. Moitra, M. Alafeef, K. Dighe, M. B. Frieman, D. Pan, Selective naked-eye detection of SARS-CoV-2 mediated by N gene targeted antisense oligonucleotide capped plasmonic nanoparticles. *ACS nano* **14**, 7617-7627 (2020).
26. T. O'Connor, S. Santaniello, B. Javidi, COVID-19 detection from red blood cells using highly comparative time-series analysis (HCTSA) in digital holographic microscopy. *Optics Express* **30**, 1723-1736 (2022).

27. K. Snyder, R. Quddus, A. D. Hollingsworth, K. Kirshenbaum, D. G. Grier, Holographic immunoassays: direct detection of antibodies binding to colloidal spheres. *Soft Matter* **16**, 10180-10186 (2020).
28. N. Goswami *et al.*, Label-free SARS-CoV-2 detection and classification using phase imaging with computational specificity. *Light: Science & Applications* **10**, 1-12 (2021).
29. C. J. Potter, Y. Hu, Z. Xiong, J. Wang, E. McLeod, Point-of-care SARS-CoV-2 sensing using lens-free imaging and a deep learning-assisted quantitative agglutination assay. *Lab on a Chip*, (2022).
30. H. Chen *et al.*, Sensitive Detection of SARS-CoV-2 Using a SERS-Based Aptasensor. *ACS Sensors* **6**, 2378-2385 (2021).
31. S. Wills, The Search for a Better COVID Test. *Optics and Photonics News* **32**, 40-47 (2021).
32. Devpost, Samsung SARS-CoV-2 detection with SERS spectroscopy research. (2020).
33. Optics.org, Research and Development. *Botanisol Analytics*, (2020).
34. Newsfile, BioMark Diagnostics Forms New Company, Bio Stream Diagnostics Inc., for '30-Second' COVID-19 Screening. *BioMark Diagnostics Inc*, (2020).
35. Ant., Advanced Nano Technologies. (2020).
36. L. Corporation, LamdaGen and COVID-19. (2020).
37. C. B. coronavirus, Surface Plasmon Resonance (SPR) for Coronavirus Research. (2020).
38. BioWorld, Todos Medical sets sights on COVID-19 test market. (2020).
39. L. Diagnostics, LightDeck: Laser Technology for Highly-Accurate, Low-Cost Diagnostics in Minutes. (2020).
40. Virolens, Virolens covid19 detection based on holography microscopy. (2021).
41. L. Morawska *et al.*, Size distribution and sites of origin of droplets expelled from the human respiratory tract during expiratory activities. **40**, 256-269 (2009).
42. M. Nicas, W. W. Nazaroff, A. J. J. o. o. Hubbard, e. hygiene, Toward understanding the risk of secondary airborne infection: emission of respirable pathogens. **2**, 143-154 (2005).
43. W. G. Lindsley *et al.*, Quantity and size distribution of cough-generated aerosol particles produced by influenza patients during and after illness. **9**, 443-449 (2012).
44. G. Johnson *et al.*, Modality of human expired aerosol size distributions. **42**, 839-851 (2011).
45. M. W. J. A. Jennison, ATOMIZING OF MOUTH AND NOSE SECRETIONS. **17**, 106 (1942).
46. P. J. Gerone *et al.*, Assessment of experimental and natural viral aerosols. **30**, 576-588 (1966).
47. R. S. Papineni, F. S. J. J. o. A. M. Rosenthal, The size distribution of droplets in the exhaled breath of healthy human subjects. **10**, 105-116 (1997).
48. K. P. Fennelly *et al.*, Cough-generated aerosols of Mycobacterium tuberculosis: a new method to study infectiousness. **169**, 604-609 (2004).
49. D. A. Edwards *et al.*, Inhaling to mitigate exhaled bioaerosols. **101**, 17383-17388 (2004).
50. S. Yang, G. W. Lee, C.-M. Chen, C.-C. Wu, K.-P. J. J. o. A. M. Yu, The size and concentration of droplets generated by coughing in human subjects. **20**, 484-494 (2007).
51. P. Fabian *et al.*, Influenza virus in human exhaled breath: an observational study. **3**, e2691 (2008).
52. C. Y. H. Chao *et al.*, Characterization of expiration air jets and droplet size distributions immediately at the mouth opening. **40**, 122-133 (2009).
