# Supplementary figures and images for: Real-time 4D tracking of airborne virus-laden droplets and aerosols
Source: Commun Eng. 2023 Jun 19;2:41. doi: 10.1038/s44172-023-00088-x (PMC10955884; doi:10.1038/s44172-023-00088-x)

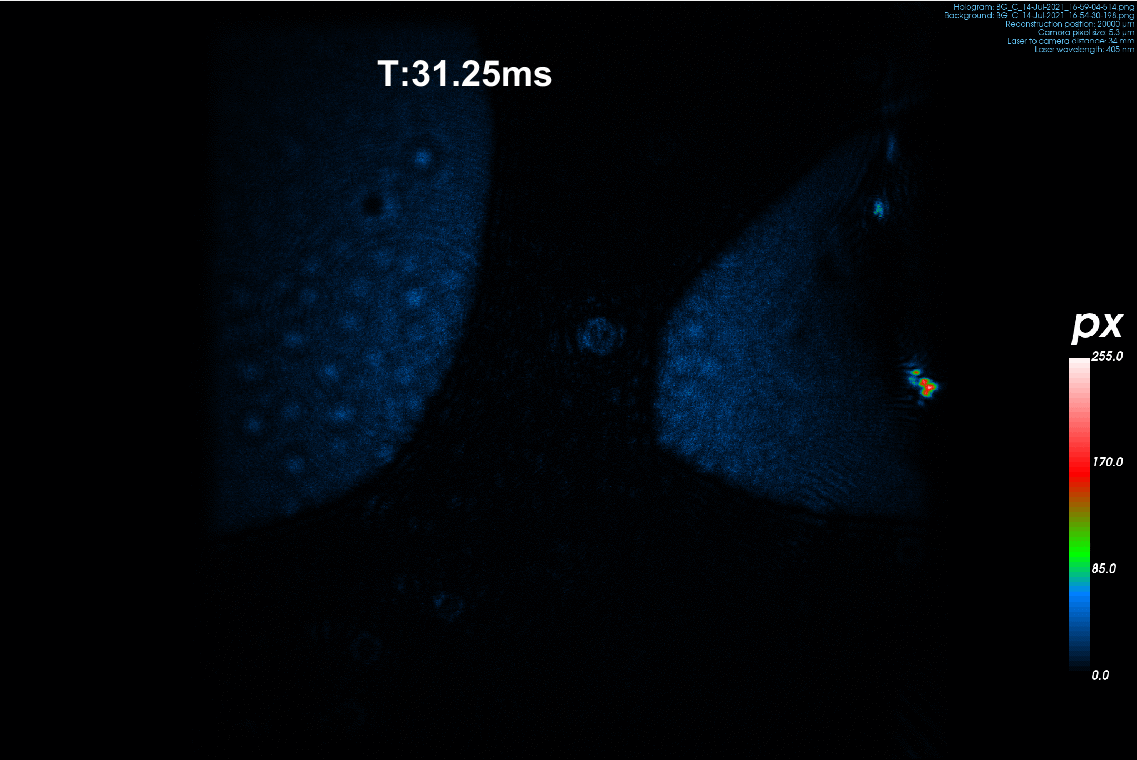

Supplement: Supplementary file 3 — Supplementary Movie S1 [file 44172_2023_88_MOESM3_ESM.gif]

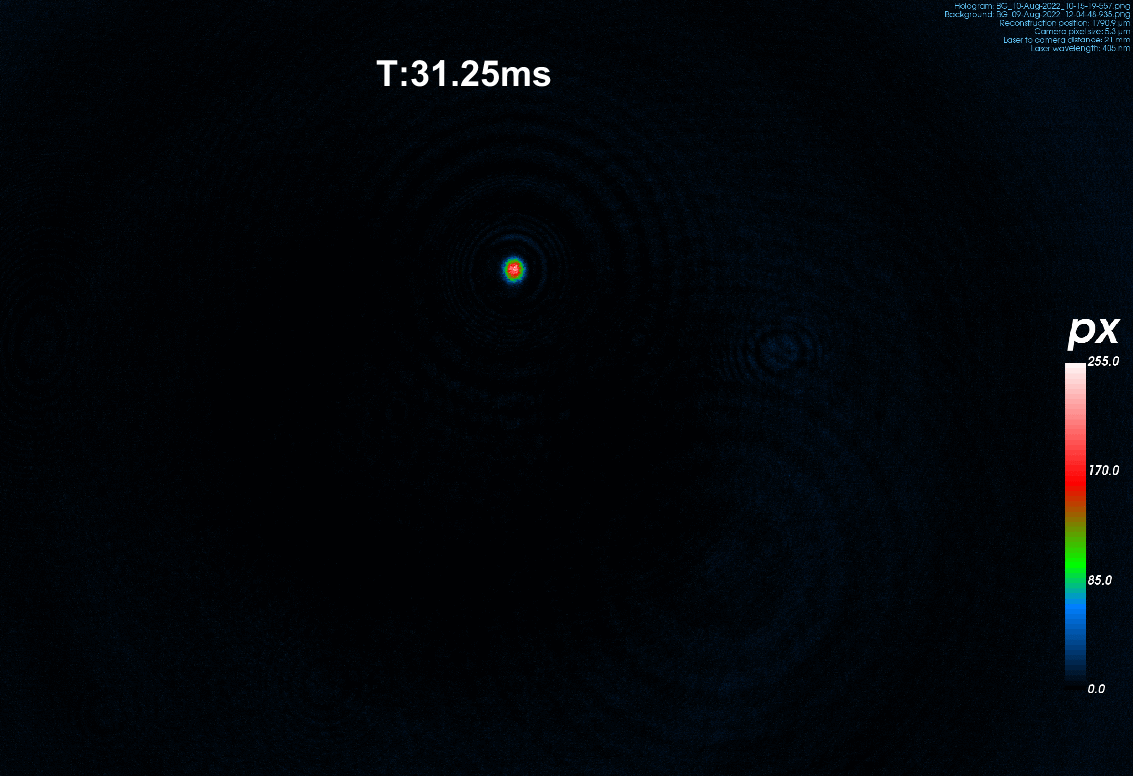

Supplement: Supplementary file 4 — Supplementary Movie S2 [file 44172_2023_88_MOESM4_ESM.gif]

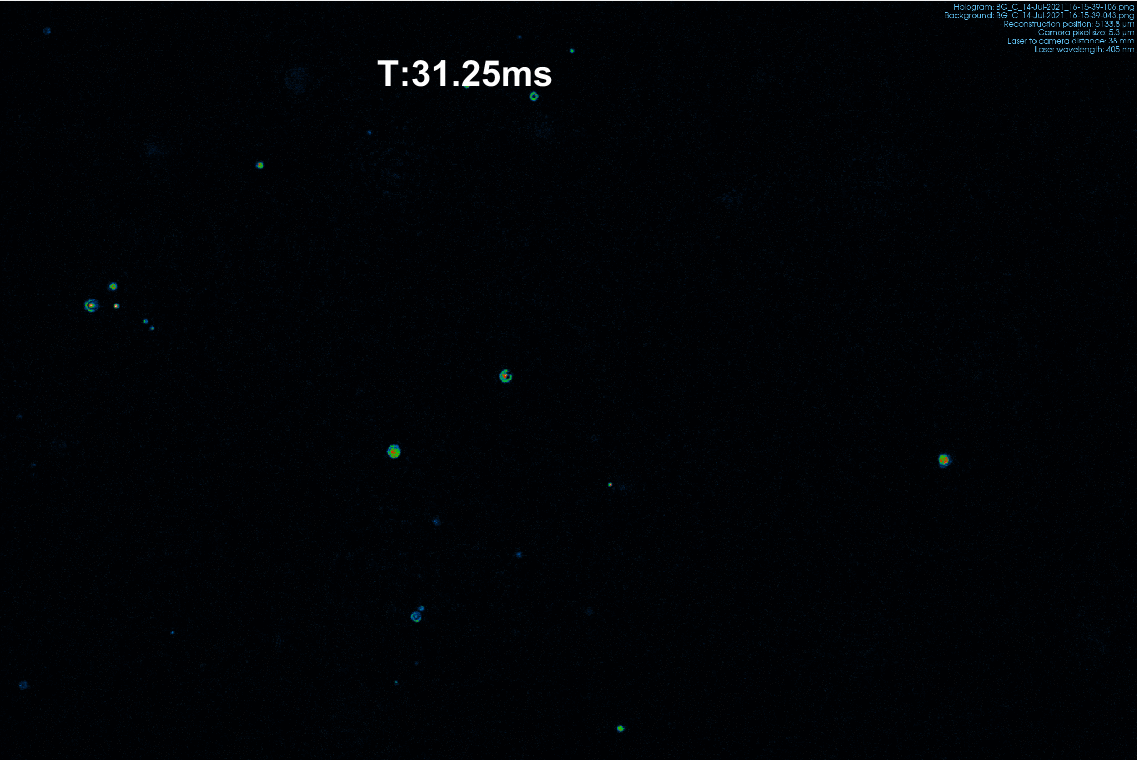

Supplement: Supplementary file 5 — Supplementary Movie S3 [file 44172_2023_88_MOESM5_ESM.gif]
